# Delay of Systemic Therapy Confers a Survival Benefit in Patients with Stage IV Non-Small-Cell Lung Cancer

**DOI:** 10.3390/cancers17213531

**Published:** 2025-10-31

**Authors:** Rike Geiken-Weinstock, Frank Griesinger, Michael Metz, Ralf Georg Meyer, Peter Staib, Tobias Overbeck, Nils Goeken, Joachim Hübner, Jörg Bäsecke

**Affiliations:** 1 Department of Health Service Research, University of Oldenburg, 26129 Oldenburg, Germany; 2 Department of Hematology and Oncology, Pius Hospital, University of Oldenburg, 26121 Oldenburg, Germany; frank.griesinger@pius-hospital.de; 3 Hematologists and Oncologists Association of Lower Saxony (NIO-Niedersachsen e.V.), 38640 Goslar, Germany; 4 Oncology Medical Practice Göttingen, 37073 Göttingen, Germany; 5 Scientific Working Group of the German Clinical Hematologists and Oncologists Association (ADHOK e.V.), 52249 Eschweiler, Germany; 6 Department of Hematology and Oncology, St. Johannes-Hospital Dortmund, 44137 Dortmund, Germany; 7 Department of Hematology and Oncology, St. Antonius Hospital Eschweiler, 52249 Eschweiler, Germany; 8 Department of Hematology and Medical Oncology, University Medical Center Göttingen, 37075 Göttingen, Germany; 9 Clinical Cancer Registry of Lower Saxony, 30625 Hannover, Germany; 10 Agency for Clinical Cancer Data of Lower Saxony (KLast), 26121 Oldenburg, Germany

**Keywords:** non-small-cell lung cancer, palliative, systemic therapy, therapy initiation, treatment delay, overall survival

## Abstract

**Simple Summary:**

We wanted to know if the timepoint of a therapy for patients with incurable lung cancer influences their survival. We observed that a later beginning is associated with better survival. By investigating many subgroups, we could rule out typical errors, e.g., a worse survival that resulted from an earlier treatment of sicker patients. Our results are important for clinical studies and patient treatment.

**Abstract:**

Background: A timely systemic therapy of patients with metastasized non-small-cell lung cancer (NSCLC) is a suggestive clinical conception. As the pre-therapeutic management is complex and includes comprehensive immunohistochemical and molecular diagnostics, the time to optimal therapy may be prolonged. Whether the timing of therapy influences the outcome still remains controversial. We investigated the therapy timing and overall survival in subgroups of NSCLC patients in the clinical cancer registry of Lower Saxony. Materials and Methods: Patients with UICC stage IV NSCLC and systemic therapy were included. Early and delayed therapy groups based on the median time from histology to therapy were defined. Median overall survival (mOS) was estimated by the Kaplan–Meier test and compared by the log rank test. Uni- and multivariate Cox regression analyses were used for independent variables. Subgroup analyses were performed according to age, ECOG-PS, metastasis stage (M1a-c) and therapy. Results: We included 1687 patients; of these, the median age was 66.8 years, and 58% of patients were male. The median time to systemic therapy was 33 days, and in our sample, 844 patients were in the early and 843 in the delayed therapy group (TG). Median overall survival of the early TG patients was 9 m vs. 14 m in the delayed TG (*p* < 0.001). Subgroup analyses confirmed consistent findings among different age, metastasis and ECOG subgroups. Conclusions: UICC IV NSCLC patients with a delayed systemic therapy had a better overall survival than those with an early therapy. This observation supports a (qualified) waiting time for systemic therapies. Therapy timing may also be a relevant confounder in clinical studies.

## 1. Introduction

A timely systemic therapy of patients with advanced non-small-cell lung cancer is a suggestive clinical conception. Today, the pre-therapeutic management of these patients is complex and includes essential procedures such as comprehensive immunohistochemical and molecular diagnostics, integration of supportive therapies and interdisciplinary decisions, meaning the initiation of an optimal cancer-specific therapy may be delayed [1,2,3,4,5,6,7,8,9]. If and how the timing of systemic therapy influences the outcome remains unclear. Some studies have found a better prognosis with a shorter interval between diagnosis and initiation of therapy [10,11,12,13]. Others have observed no association, and some indicate that an early initiation of therapy is associated with a worse prognosis [14,15,16,17,18,19,20,21]. In most studies, confounding factors that might have an influence on the outcome were not accounted for, such as age, gender, the ECOG score and the type of therapy, especially targeted and immunotherapy. We therefore investigated the effect of therapy timing on overall survival in multiple subgroups of NSCLC patients in a German cancer registry.

## 2. Materials and Methods

In this retrospective study, data were obtained from the Lower Saxony clinical cancer registry (Klinisches Krebsregister Niedersachsen, KKN). Anonymized data from patients who were diagnosed with UICC stage IV NSCLC between July 2018 and June 2021 were included after giving informed consent and as approved by the ethics committee of the University Medicine Oldenburg (UMO, Az: 2022-080) on 19 May 2022.

Only patients with palliative systemic therapies were included. The early and delayed therapy groups (TGs) were separated by the median time from histologically confirmed diagnosis to first systemic treatment. Median overall survival was estimated using the Kaplan–Meier test and compared by the log rank test. Uni- and multivariate Cox regression analysis was used for independent variables. Subgroup analyses were performed according to age, ECOG-PS (of 0, 1, ≥2), distant metastasis status (M1a–c) and type of therapy (chemotherapy, immunotherapy, combined chemoimmunotherapy, targeted therapy, additive therapies, other combined therapies). Additive therapies refer to systemic treatments that are not considered specific anticancer therapies. Examples include the use of corticosteroids or bisphosphonates. *p*-values < 0.05 were considered statistically significant.

## 3. Results

### 3.1. Patient Characteristics

This study included 1687 patients. The median age was 66.8 years, and 58% of patients were male. Most patients received chemotherapy (33.2%) or combined immunochemotherapy (33.1%). Immunotherapy alone was administered in 19.8% of cases and targeted therapy in 6.3%. Additive therapies (e.g., cortisone) were applied in 4.7% of patients, and 2.9% received other combined therapies. Among the sample, 22.7% had an ECOG-PS of 0, 17.6% an ECOG-PS of 1, 5% an ECOG-PS of 2 or higher and, in 57.7%, the ECOG-PS was unknown. The median time from histologically confirmed diagnosis to first systemic therapy was 33 days (mean = 49.9 days, 95% CI 44.7–51.1), and 844 patients received systemic therapy within (early TG) and 843 patients beyond this median (delayed TG). Patients were equally distributed concerning age, gender and ECOG-PS. In contrast, the distributions of the types of metastasis stages and types of therapy were significantly different. M1a patients were more present in the delayed than in the early TG (26.3% vs. 20.1%), whereas M1c patients were more present in the early than in the delayed TG (48.3% vs. 55.5%). In the early TG, more patients received only chemotherapy (37% versus 29.4%) or additive therapies (7.6% versus 1.5%). Immunotherapies (17.8% versus 22%) and immunochemotherapies (28.3% versus 37.8%) were more frequently administered in the delayed TG, and targeted therapies were equally distributed in both therapy groups (Table 1).

### 3.2. Survival Uni- and Multivariate Regressions

**Figure 1:**
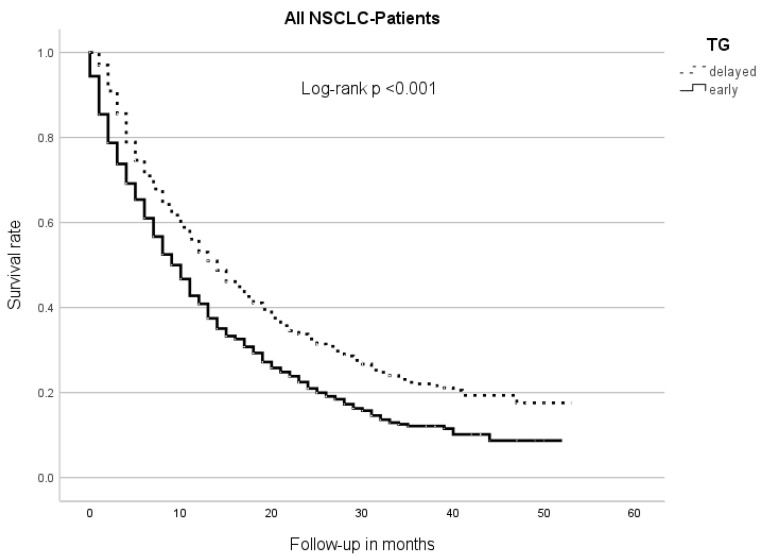
Median overall survival between therapy groups (TGs). Patients of the early TG had a significantly shorter overall survival than those of the delayed TG.

The median overall survival (OS) of the early TG patients was 9 m (95% CI 8.0–10.0), while the delayed TG patients had an mOS of 14 m (95% CI 12.5–15.5, *p* < 0.001) (Figure 1).

The univariate Cox regression analysis showed that a higher age (HR 1.02, 95% CI 1.01–1.03, *p* = 0.01), ECOG-PS of 1 (HR 1.29, 95% CI 1.08–1.54, *p* =0,004) or ≥2: (HR 2.56, 95% CI 1.98–3.31, *p* < 0.001), distant metastasis stage M1c (HR 1.41; 95% CI 1.22–1.62) and receiving additive therapies (HR 1.34, 95% CI 1.04–1.74, *p* = 0.03) were significantly associated with a worse survival. In contrast, patients in the delayed TG (HR 0.70, 95% CI 0.62–0.78, *p* = 0.001), patients with a longer period between diagnosis and therapy initiation (HR 0.99; 95% CI 0.996–0.998, *p* < 0.001) and patients who received mono-immunotherapy (HR 0.82; 95% CI 0.70–0.96, *p* = 0.01), immunochemotherapy (HR 0.81; 95% CI 0.71–0.93, *p* = 0.002) or targeted therapy (HR 0.40; 95% CI 0.30–0.53, *p* < 0.001) had a superior survival. In the multivariate analysis, the delayed TG (HR 0.77; 95% CI 0.62–0.93, *p* = 0.007) and targeted therapy (HR 0.45; 95% CI 0.30–0.67, *p* < 0.001) were still associated with a significantly better survival after backward selection. The survival was inferior in elderly patients (HR 1.02; 95% CI 1.01–1.03, *p* = 0.002), an ECOG-PS of 1 (HR 1.26; 95% CI 1.05–1.51, *p* = 0.01) or ≥2 (HR 2.19; 95% CI 1.68–2.86, *p* < 0.001), metastasis stage M1c (HR 1.42; 95% CI 1.15–1.75, *p* = 0.001) and additive therapies (HR 1.47; 95% CI 1.05–2.05) (Table 2).

### 3.3. Survival in Subgroups

Separate analyses of the subgroups that influenced mortality in the multivariate Cox regression were performed. Three age groups based on their percentiles (<60 years; 60–74 years; ≥75 years) were investigated. Patient age affected mOS. Patients <60 y had an mOS of 14 m (95% CI 11.70–16.30), patients in the 60–74 y group had an OS of 12 m (95% CI 10.79–13.21) and patients >75 years had an mOS of 9 m (95% CI 7.69–10.32, *p* <0.001). In each of these age groups, the early TG again had a significantly shorter mOS than the delayed TG. Patients<60 y in the early TG had an mOS of 11 m (95% CI 9.10–12.90). The mOS of the delayed TG patients was 18 m (95% CI 14.23–21.77; *p* = 0.002). In the age group 60–74 y, an mOS of 10 m was observed for the early TG (95% CI 8.63–11.37) and 14 m for the delayed TG (95% CI 11.67–16.34; *p* <0.001). In the age group ≥75 years, the mOS of the early TG was 7 m (95% CI 5.38–8.62), and that of the delayed TG was 11 m (95% CI 8.68–13.32; *p* < 0.001) (Supplementary Figure S1).

Concerning the metastasis stages, the mOS in the M1a patients was 15 m (95% CI 12.13–17.87), it was 13 m in M1b (95% CI 11.41–14.59), and it was 9 m in M1c patients (95% CI 7.96–10.04; *p*-value < 0.001). In stage M1a, the mOS of the delayed TG was 18 m (95% CI 14.64–21.36) and that of the early TG 13 m (95% CI 10.14–15.86; *p* = 0.005). In stage M1b, the mOS of the delayed TG (15 m; 95% CI 12.36–17.64) was also significantly longer than that of the early TG (12 m; 95% CI 10.41–13.59; *p* = 0.038) as well as in stage M1c (delayed TG: 12 m, 95% CI 10.02–13.98; early TG: 7 m; 95% CI 5.82–8.18, *p* < 0.001) (Supplementary Figure S2).

**Figure 2:**
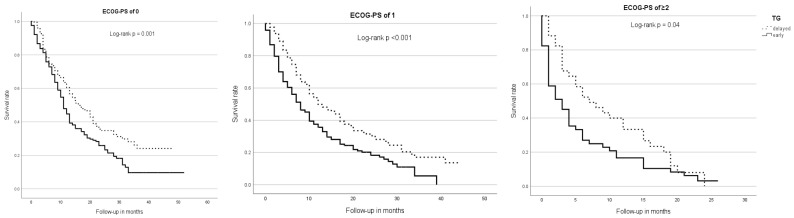
Median overall survival, therapy group, and ECOG-PS. Across all ECOG subgroups, patients of the early TG showed a significantly shorter overall survival than those of the delayed TG.

Regarding ECOG subgroups, patients with an ECOG-PS of ≥2 (mOS = 4 m; 95% CI 2.43–5.57) had a significantly shorter survival time than patients with an ECOG-PS of 0 (mOS = 13 m; 95% CI 11.3–14.7) or 1 (mOS = 10 m; 95% CI 8.34–11.66, *p* < 0.001). In all ECOG subgroups, patients of the early TG had a significantly shorter mOS than patients of the delayed TG—ECOG 0: 11 m (95% CI 9.52–11.48) vs. 16 m (95% CI 12.17–19.83; *p* = 0.001), ECOG 1: 8 m (95% CI 6.17–9.83) vs. 12 m (95% CI 7.79–16.21; *p* < 0.001) and ECOG ≥2: 3 m (95% CI 1.26–4.74) vs. 7 m (95% CI 2.58–11.43; *p* = 0.04) (Figure 2).

Concerning the therapy groups, targeted therapy conferred the best mOS (28 m; 95-CI 18.29–37.71), followed by immunotherapy (12 m; 95-CI 9.88–14.12), immunochemotherapy (12 m; 95% CI 10.73–13.27), other combinations (11 m; 95% CI 7.21–14.79) and chemotherapy (9 m; 95% CI 7.86–10.14). The shortest OS was observed with additive therapies (4 m; 95% CI 2.11–5.89, *p* < 0.001). The mOS was also different within these therapy groups, dependent on the early or late TG. In the chemotherapy group, the mOS of the early TG was 8 m (95% CI 6.71–9.29) and that of the delayed TG was 10 m (95% CI 7.67–12.33; *p* < 0.001). Patients of the early TG also showed a significantly shorter mOS in the immunotherapy group (11 m; 95% CI 8.53–13.47 vs. 14 m; 95% CI 10.07–17.93; *p* = 0.01). The same was true in the immunochemotherapy group (11 m; 95% CI 9.51–12.49 vs. 14 m; 95% CI 11.67–16.33; *p* = 0.001) and for additive therapies (2 m; 95% CI 0.86–3.14 vs. 15 m; 95% CI 10.0–19.0; *p* = 0.029). A trend was observed in patients receiving targeted therapy (25 m; 95% CI = 13.29–36.71 vs. 28 m; 95% CI = 14.85–41.15) and other combination therapies (8 m; 95% CI = 2.03–13.97 vs. 13 m; 95% CI = 9.93–16.07) but was not statistically significant (Supplementary Figure S3).

In addition, a consolidated ECOG subgroup (ECOG PS 0 and ≥1) and therapy type analysis (chemotherapy-based, immunotherapy and targeted therapy) was performed to confirm that the observed results in the therapy subgroups were present in both ECOG subgroups. In the chemotherapy-based and ECOG-PS 0 subgroup (*n* = 255), a significantly shorter survival time in the early TG (11 m; 95% CI = 9.63–12.37) was observed compared to the delayed TG (15 m; 95% CI = 10.88–19.13; *p* = 0.013). The chemotherapy-based, ECOG-PS of ≥1 subgroup (*n* = 250) also had a worse mOS of 7 m (95% CI = 4.86–9.14), compared to 11 m (95% CI = 8.20–13.80; *p* = 0.006) in the delayed group. Patients who received immunotherapy and had an ECOG-PS of 0 (*n* = 73) showed a trend towards a worse mOS in the early TG (15 m; 95% CI = 4.32–25.68 vs. 20 m; 95% CI = 9.05–31.00). However, this difference was not statistically significant (*p* = 0.129). The mOS of immunotherapy patients with an ECOG-PS of ≥1 (*n* = 72) in the early TG was 8 m (95% CI = 4.20–11.80), compared to 7 m (95% CI = 3.27–10.73), and also not statistically significant (*p* = 0.538). The mOS of patients with targeted therapy and an ECOG-PS of 0 (*n* = 33) did not differ between both TGs (early TG 25 m; 95% CI 11.51–38.49 vs. delayed TG 22 m; 95% CI 9.28–34.72; *p* = 0.879). The same result was observed in the cohort with an ECOG-PS ≥1 (*n* = 20; early TG 20 m; 95% CI = 0.00–52.14 vs. delayed TG 34 m; 95% CI = 3.40–54.61; *p* = 0.290).

## 4. Discussion

In this cancer registry data analysis, we investigated if the timing of systemic therapy in patients with UICC stage IV non-small-cell lung cancer influences overall survival, as suggested by some but not all previous studies. Further patient and therapy-related parameters that modulate survival were determined by the analysis of various subgroups. In addition, real-world data concerning epidemiology and the type of palliative therapy were identified. In general, about two-thirds of all patients received either chemotherapy or immunochemotherapy, about 20% received immunotherapy only and 6% targeted therapy. In the delayed therapy group, more patients were treated with immuno- and immunochemotherapy. In the early therapy group, more patients received chemotherapy, whereas the targeted therapy was equally distributed between the early and delayed groups.

Our key finding was that patients of the early therapy group (TG) had a significantly worse OS compared to patients of the delayed TG (9 m vs. 14 m). In addition, this finding was confirmed in almost any subgroup. The association of a worse OS in unselected patients of the early TG is suggestive as those with a higher ECOG-PS were more present in this group. The early TG also included more patients with multiple distant metastasis (M1c). Both criteria increase mortality [21,22,23] and may prompt an early therapy by a “sicker-quicker” clinical decision [20,24,25,26]. To sort out this bias, ECOG-matched subgroups (ECOG-PS of 0, 1 and ≥2) were analyzed. Here, our previous findings were confirmed in each ECOG subgroup [18]. The difference was most prominent in the ECOG ≥2 group. Here, the mOS of patients with an early therapy was more than 50% shorter than of those in the delayed group. Further details such as a comorbidity index that would allow us to discriminate between imminent and variable influences on the ECOG score (e.g., comorbidities and the disease itself) were not available. We then looked at possible confounders, such as the stage of distant metastasis, that may worsen the OS but not necessarily the initial ECOG score. We indeed observed more patients with a higher stage in the early group, which probably reflects clinical decisions as mentioned above. But in the subgroup analysis, an early therapy was inferior in each of the metastasis stages, which was confirmed in the multivariate Cox regression. So, again, this bias was not responsible for the association of an early therapy with a worse survival.

Many therapeutic regimens for NSCLC are available today that improve survival and reduce side effects but may prolong therapy initiation due to necessary immunohistochemical and molecular diagnosis [27]. Chemotherapy was more frequently administrated in the early group. The disadvantage of an early therapy remained significant when the chemotherapy, immunotherapy and the combined immunochemotherapy subgroups were analyzed. More data concerning the type of chemo-, immune- or targeted therapy were not available. The patients with targeted therapy were an exception: only a trend towards a worse mOS in the early vs. delayed TG (25 m vs. 28 m) was observed. Due to the characteristics of targeted therapy, disadvantages of an early and advantages of a delayed therapy may be less relevant than with other regimens [28,29,30]. This observation also does not support “bridging” chemotherapy-based therapies, which are applied before the molecular diagnosis has been completed. We then performed a combined subgroup analysis that included the type of therapy and the ECOG-PS (0 and ≥1). A worse OS was observed in patients of the early TG in each ECOG subgroup who received a chemotherapy-based regimen, so we could also reproduce our results in this combined analysis. Patients with immunotherapy or targeted therapy showed no significant OS differences in the early and delayed TGs. This may be due to side effects of the chemotherapy-based regimen that increased mortality in patients with a worse ECOG-PS. Patients of the delayed TG may have received supportive therapies in the meantime, which may have led to a survival benefit, but those therapies are not documented in the cancer registry.

## 5. Conclusions

An early initiation of a chemo- or immunochemotherapy may be associated with a worse prognosis in UICC stage IV NSCLC patients. Whether supportive therapies or other parameters are responsible for the survival benefit of patients with a later therapy initiation remains unclear. The prognosis of patients who receive targeted therapies seems to be less dependent on timing, probably as they experience fewer side effects that influence morbidity. “Bridging” chemotherapies prior to the final immunohistochemical and molecular results are not supported by these findings. Our results also indicate that the timing of therapy may be a relevant parameter in clinical studies. As our results are based on a retrospective cancer registry analysis, we want to emphasize that practice changes require prospective trials.

## Figures and Tables

**Table 1:** Patient characteristics.

Characteristics	All Patients	Early TG	Delayed TG	*p*-Value
Total, *n*	1687	844	843	
Mean age, years (min;max)	66.8 (26;94)	66.8 (26;94)	66.7 (32;88)	0.72
Gender, *n* (%)				
Male	980 (58.1)	487 (57.7)	493 (58.5)	
Female	707 (41.9)	357 (42.3)	350 (41.5)	0.75
ECOG-PS, *n*(%)				
0	383 (22.7)	204 (24.2)	179 (21.2)	
1	297 (17.6)	168 (19.9)	129 (15.3)	
≥2	85 (5)	51 (6.1)	34 (4)	0.45 ^1^
Unknown	922 (54.7)	421 (49.9)	501 (59.4)	
Distant metastasis (stage cM), *n* (%)				
1a	392 (23.2)	170 (20.1)	222 (26.3)	
1b	400 (23.7)	195 (23.1)	205 (24.3)	
1c	875 (51.9)	468 (55.5)	407 (48.3)	0.01 ^1^
Unknown	20 (1.2)	11 (1.3)	9 (1.1)	
Therapy type, *n* (%)				
Chemotherapy	560 (33.2)	312 (37)	248 (29.4)	
Immunotherapy	335 (19.8)	150 (17.8)	185 (22)	
Targeted therapy	106 (6.3)	52 (6.2)	54 (6.4)	
Additive therapies	79 (4.7)	66 (7.8)	13 (1.5)	
Immunochemotherapy	558 (33.1)	239 (28.3)	319 (37.8)	
All other combinations	49 (2.9)	25 (2.9)	24 (2.9)	<0.001

^1^ Missing values excluded.

**Table 2:** Uni- and multivariate regressions.

Factor	Univariate		Multivariate	
	HR (95-CI)	*p*-Value	HR (95-CI)	*p*-Value
Age (years)	1.02 (1.01–1.03)	<0.001	1.02 (1.01–1.03)	0.002
Gender				
Male	Ref.			
Female	0.91 (0.81–1.02)	0.09		
Therapy group				
Early	Ref.			
Delayed	0.70 (0.62–0.78)	<0.001	0.77 (0.62–0.93)	0.007
Time to treatment, days	0.99 (0.996–0.998)	<0.001	1.00 (0.99–1.00)	0.07
ECOG-PS ^1^				
0	Ref.			
1	1.29 (1.08–1.54)	0.004	1.26 (1.05–1.51)	0.01
≥2	2.56 (1.98–3.31)	<0.001	2.19 (1.68–2.86)	<0.001
Distant metastasis (stage cM) ^1^				
1a	Ref.			
1b	1.09 (0.92–1.28)	0.33	1.13 (0.88–1.46)	0.34
1c	1.41 (1.22–1.62)	<0.001	1.42 (1.15–1.75)	0.001
Therapy type				
Chemotherapy	Ref.			
Immunotherapy	0.82 (0.70–0.96)	0.01	0.86 (0.67–1.10)	0.22
Immunochemotherapy	0.81 (0.71–0.93)	0.002	0.84 (0.68–1.03)	0.09
Targeted therapy	0.40 (0.30–0.53)	<0.001	0.45 (0.30–0.67)	<0.001
Additive therapies	1.34 (1.04–1.74)	0.03	1.47 (1.05–2.05)	0.02
All other combinations	0.92 (0.66–1.28)	0.62	1.47 (0.81–2.65)	0.21

HR = hazard ratio; CI = confidence interval; ^1^ missing values excluded.

## Data Availability

Original data are available from the corresponding author upon request.

## Supplementary Materials

The following supporting information can be downloaded at https://www.mdpi.com/article/10.3390/cancers17213531/s1, Figure S1: Median overall survival, therapy group and age groups; Figure S2: Median overall survival, therapy group and distant metastases; Figure S3: Median overall survival, therapy group and therapeutic subgroups.
