# Supplementary material for: Delay of Systemic Therapy Confers a Survival Benefit in Patients with Stage IV Non-Small-Cell Lung Cancer
Source: Cancers (Basel). 2025 Oct 31;17(21):3531. doi: 10.3390/cancers17213531 (PMC12609030; doi:10.3390/cancers17213531)
Supplement: Supplementary file 1 [file cancers-17-03531-s001.zip › cancers-3940016-supplementary.pdf]

Supplemental Figures

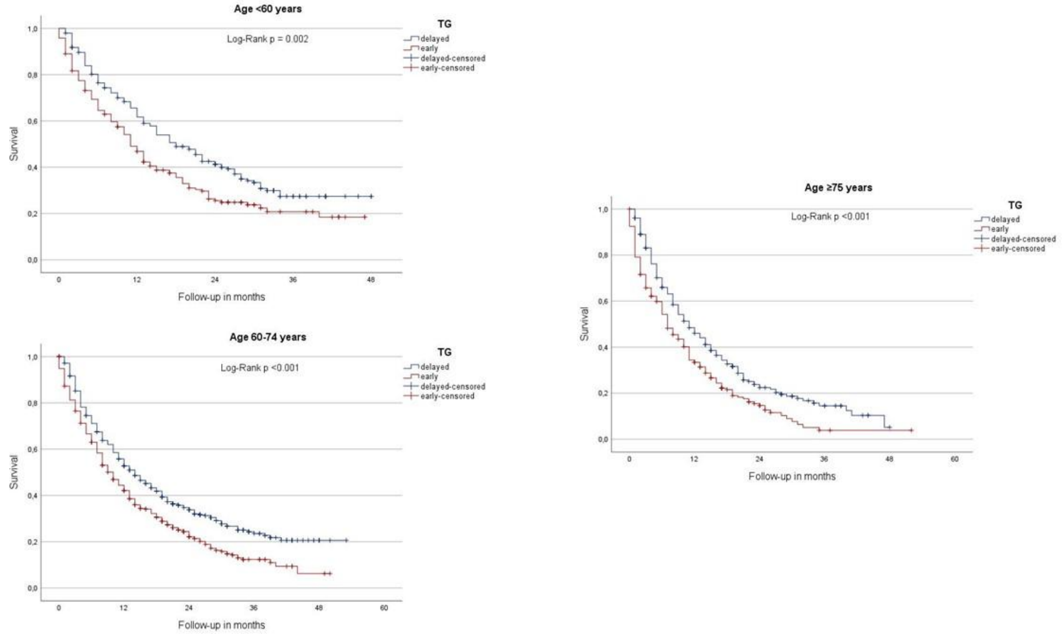

Figure S1. Median overall survival, therapy group and age groups. In each age group, patients of early TG showed a significantly shorter overall survival than those of delayed TG.

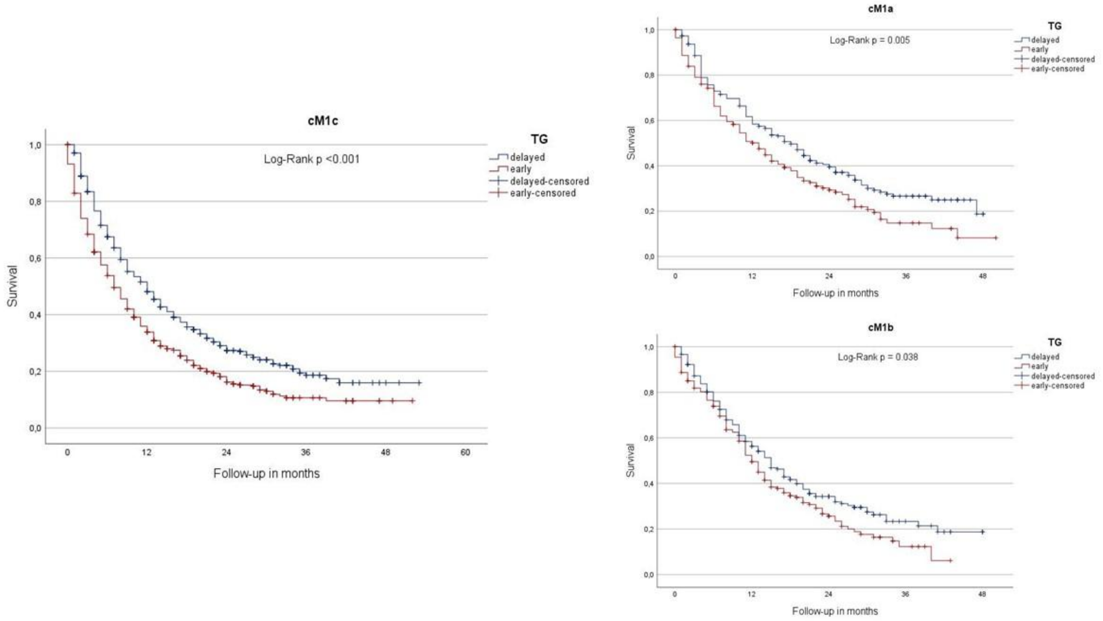

Figure S2. Median overall survival, therapy group and distant metastases. In all stages (M1a - M1c), patients of early TG showed a significantly shorter overall survival than those of delayed TG.

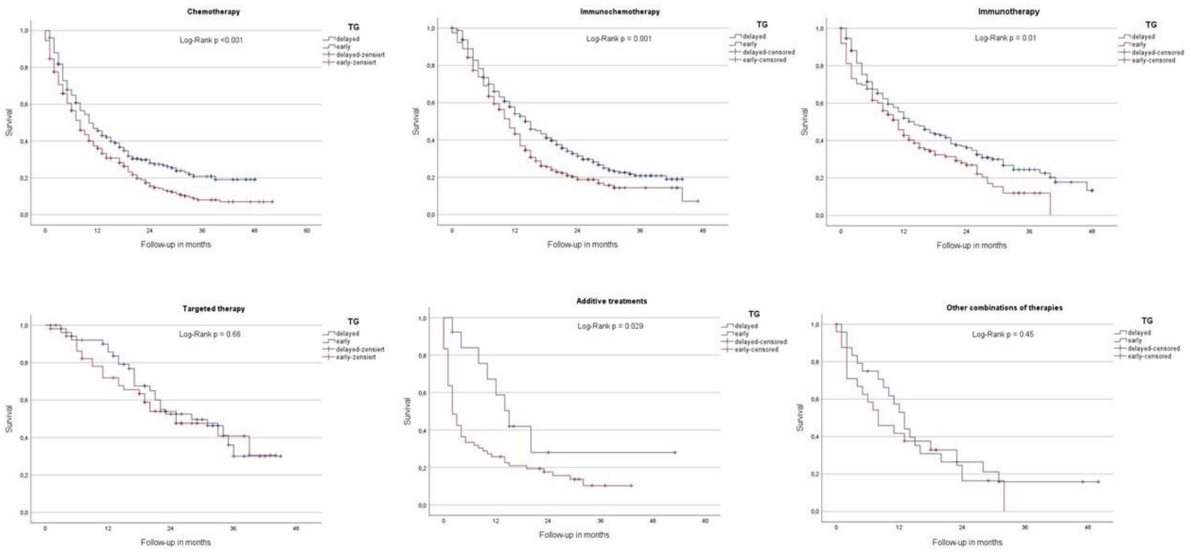

Figure S3. Median overall survival, therapy group and therapeutic subgroups. A significantly shorter overall survival of patients of early TG was observed for chemotherapy, immunochemotherapy, immunotherapy and additive therapy.
